# Transsphenoidal Optic Canal Decompression for Traumatic Optic Neuropathy Assisted by a Computed Tomography Image Postprocessing Technique

**DOI:** 10.1155/2020/1870745

**Published:** 2020-08-12

**Authors:** J. Li, Q. S. Ran, B. Hao, X. Xu, H. F. Yuan

**Affiliations:** ^1^Department of Ophthalmology, Daping Hospital, Army Medical University, Chongqing 400042, China; ^2^Department of Radiology, Daping Hospital, Army Medical University, Chongqing 400042, China; ^3^Department of Stem Cell and Regenerative Medicine, State Key Laboratory of Truma, Burn and Combined Injury, Daping Hospital, Army Medical University, Chongqing 400042, China

## Abstract

The endoscopic transethmoidal approach is favored for the lack of external scars, a wide field of view, and rapid recovery time. But the effect of iatrogenic trauma should not be ignored due to the removal of the uncinate process and anterior and posterior ethmoidal sinus. Anatomically, the optic nerve is close to the sphenoid sinus and Onodi cell. In order to preserve the uncinate process and ethmoidal sinus, we perform endoscopic transsphenoidal optic canal decompression (ETOCD), which is less invasive. However, the anatomy of sphenoid sinus is quite variable, and the anatomical landmarks are rare. Therefore, identifying the position of optic canal is particularly important during surgery. To solve this, we use a postprocessing technique to identify the position of the optic nerve and internal carotid artery on the sphenoid sinus wall. Our results find that VA in 13 patients improved, with a total improve rate of 59.1%. No serious complications were found. We also found that the length of optic canal is different and the medial wall of the optic canal was the longest (*p* < 0.05). The middle section of the optic canal is the narrowest, which was significantly different from cranial mouth and orbital mouth (*p* < 0.05). We assumed that decompression may not require removal of all medial wall. If we remove the length of the shortest wall on the medial wall of the optic canal, the compression may be relieved. Thus, ETOCD was a feasible, safe, effective, and less-invasive approach for patients with TON. The CT postprocessing imaging facilitated recognition of the optic canal during surgery. The decompression length of the medial wall may not need to be completely removed, especially near the cranial mouth.

## 1. Introduction

Traumatic optic neuropathy (TON) is rare but a serious complication of head trauma, resulting in partial or complete loss of vision [1, 2], and it can be caused by direct or indirect optic nerve injury. Direct injury has a poor prognosis; however, indirect injuries, such as edema and hematoma, may benefit from treatment [3]. Optic canal decompression is a method used to treat indirect TON, which enables more room for expansion of the traumatized nerve, thus limiting the secondary optic nerve injury. Compared to a lateral or medial orbitotomy, as well as transcranial approaches, the endoscopic transethmoidal offers many advantages, including a wide field of view, rapid recovery time, and more acceptable cosmetic results without external scars. However, the effect of iatrogenic trauma should not be ignored due to removal of the uncinate process and the anterior and posterior ethmoidal sinus [4, 5]. Anatomically, the optic canal is immediately superolateral to the sphenoid sinus, composed of the lateral surface of the sphenoid body and parts of the lesser sphenoid wing. Considering the anatomical position, some researchers have modified the endonasal approach through a single endoscopic transsphenoidal approach, which is less invasive [6, 7]. However, the anatomy of sphenoid sinus is quite variable and the anatomical landmarks are rare. Moreover, with the anatomy disorders caused by the fracture of the skull base, finding the optic canal during surgery becomes more difficult. Therefore, identifying the position of optic canal is particularly important during surgery.

The computed tomography (CT) postprocessing imaging technique is one of the several computer-assisted technologies that have been widely used in surgical procedures and in preoperative planning or postoperative assessment [8–10]. Virtual reality (VR) represents a contemporary standard three-dimensional (3D) image postprocessing technique, and it is mainly used to visualize complex anatomical information and clear spatial relationships between and among different tissues [11, 12]. Therefore, during surgery, it can facilitate recognition of the optic canal and internal carotid. In the present research, we aimed to provide more evidence, including effectiveness and safety, supporting the endoscopic transsphenoidal approach to treat TON, and introduce a technique that could help recognize the optic nerve and its relationship with the internal carotid during surgery.

## 2. Materials and Methods

### 2.1. Patients

All cases of indirect TON encountered at the authors' department between December 1, 2014, and January 1, 2017, were retrospectively reviewed. All patients underwent endoscopic transsphenoid optic canal decompression (ETOCD) after intravenous treatment with methylprednisolone, with no improvement in visual acuity (VA). Ethical approval was obtained ([2014]004) from the Medical Ethics Committee of the authors' hospital. The study involved 22 patients, of whom 1 was female and 21 were male. No control group comparisons were made. The average age at the time of injury was 28.9 years (range, 12 to 64 years), with surgery performed 5 to 67 days after injury. Indications for ETOCD were as follows: VA <0.1, with no improvement after intravenous treatment of methylprednisolone; partial or complete visual loss, with a history of head trauma and without eye injury, and CT revealing no definite fracture in the optic canal; CT revealing fracture with compression in the bony optic canal; and preoperative VEP scan revealing prolonged absolute latency or amplitude reduction. The data used to support the findings of this study are included within the article.

### 2.2. CT and Postprocessing

All data were collected from the department of radiology of the hospital. CTA images were acquired using a 64-slice multidetector scanner (Sensation 64, GE Healthcare, Madison, WI, USA). Imaging parameters for CT scanning and detailed procedures for postprocessing are listed in the supplementary materials. Thin-slice CT transverse images from all subjects were first uploaded to the picture archiving and communication system, with the CT data (Digital Imaging and Communications in Medicine 3.0) then inputted into a computer-aided clinical research platform using aw4.6 software (GE Healthcare) to analyse the images. The 3D structure of each of the sphenoid sinus bones was reconstructed transparently and fused with the 3D image of the optic nerve and the internal carotid-ophthalmic artery, using transparency, colour, and shading to enable better representation of the position and relationship of the optic nerve and internal carotid on the skull base.

### 2.3. Surgical Procedure

**Figure 1:**
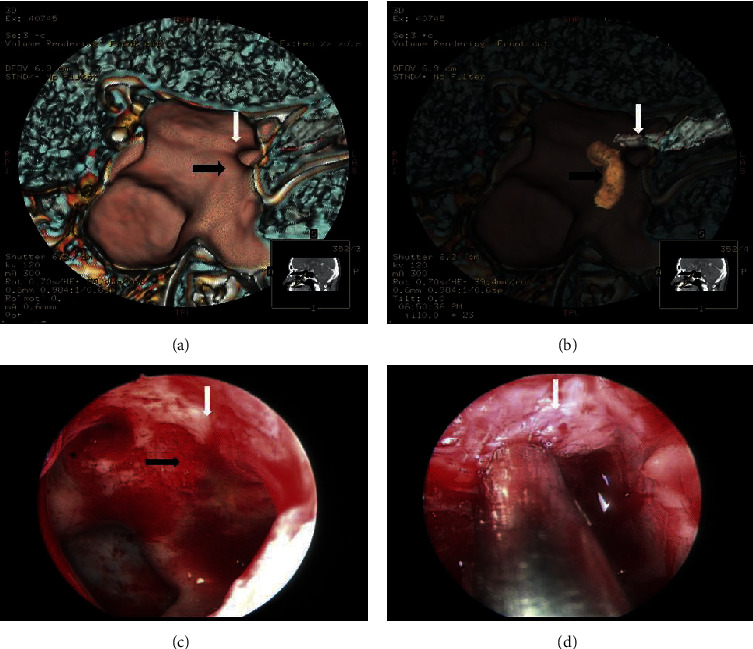
A 42-year-old man with traumatic optic neuropathy. The VR image and endoscopic image of optic nerve and internal carotid artery present on the inner wall of sphenoid sinus. White arrow shows optic canal; black arrow shows internal carotid artery. (a) The VR image of the medial wall of the sphenoid sinus. (b) The fuse VR image of the medial wall of the sphenoid sinus, the optic nerve, and the internal carotid artery. (c) The anatomical structure of the medial wall of the sphenoid sinus under endoscopy. (d) The optic nerve under endoscopy.

Patients were prepared in a routine manner for endoscopic sinus surgery under general anesthesia. Cotton swabs were soaked in 1 : 100000 epinephrine solution and placed in the nasal cavity to ensure vasoconstriction. The superior turbinate was removed to expose the ostium of the sphenoid sinus. The sphenoid sinus was opened, and the posterior ethmoids were slightly opened; the ostium was enlarged to the lateral wall, and the optic nerve canal can be identified. Without a neuronavigation, we use the preoperative CT postprocessing imaging (Figures 1(a) and 1(b)) combined with anatomical marks to confirm the position of the optic canal and the internal carotid artery. The medial wall of the optic canal was thinned using a microdrill. The length was fixed according to the preoperative CT measurement; the width is approximately one-half of the cross-sectional diameter of the optic canal, and the optic nerve sheath is incised at multiple points using a sharp 9# MVR scalpel. Finally, the operating field of the optic canal was covered using a piece of sterile gelatin sponge that was immersed in triamcinolone acetonide (50 mg/mL).

### 2.4. Statistical Analysis

Statistical analyses were performed with SPSS version 17.0 (IBM Corporation, Chicago, IL, USA). The VA improve rate was defined as the number of patients with improved VA divided by the overall number of patients who underwent ETOCD. Multiple comparisons were used to compare the length or circumference of the optic canal. Differences with *p* ≤ 0.05 were considered to be statistically significant.

## 3. Results

Flash visual evoked potential (VEP) revealed amplitude reduction in 19 patients; the latency period of the P100 wave was prolonged in 5 cases, and the P2 wave was prolonged in 14 cases. Pattern VEP in 1 case revealed the amplitude reduction, and the latency of each wave was prolonged. Flash VEP failed to induce a wave form in 2 cases. The time interval for 13 cases was from 5 to 21 days, and their retinal nerve fiber layer (RNFL) was normal. Nine cases were >21 days, and their RNFL exhibited varying degrees of thinning. All other patient clinical data are summarized in Table 1.

ETOCD was successfully performed in all 22 patients, in whom surgery was guided by the postprocessing imaging technique, which successfully identified the optic canal during surgery. Among the 22 patients, the eyesight of three reached 0.1-0.2 postoperatively and two regained light sensation. The eyesight of two patients could see hand motion, and one could count fingers. Five patients reached 0.02–0.06. VA in 13 patients improved, with a total improvement rate of 59.1%. Seven patients, however, still had no light sensation. Patients with no light perception, light perception, hand motion, and finger count exhibited VA improvement of 22.2%, 25%, 20%, and 66.7%, respectively. One patient developed cerebrospinal fluid leakage. No other severe complications were observed.

To determine which wall was the longest or shortest, the length of optic canal walls was measured. The medial wall of the optic canal was the longest, which has significant difference to the superior wall, lateral wall, and inferior wall (*p* < 0.05) (Table 2).

For better decompression of the optic canal, the pipe size of the optic canal was measured. The middle section of the optic canal is the narrowest, which was significantly different from the cranial mouth and orbital mouth (*p* < 0.05) (Table 3).

## 4. Discussion

The most widely accepted treatments for TON have traditionally involved observation, high-dose steroid therapy, and surgical decompression. Unfortunately, however, the optimal treatment remains controversial, with none proving definitive conclusions. A recent meta-analysis reported that steroids are of questionable benefit and may be harmful. The visual improvement rate of conservative treatment was 40%–60%. However, the results may be based on good initial VA and would more likely to be treated conservatively [12]. According to previous research, the VA improvement rate of ETOCD is 20%–58%. Patients with no light perception, light perception, hand motion, and finger counting demonstrated response rates to endoscopic endonasal surgery of 41%, 89%, 93%, and 84%, respectively [13]. These results imply that patients with good preoperative VA compared to those with complete blindness may achieve better prognosis. Regarding surgical timing, some researchers have found that early treatment may yield better VA results. Xie reported that surgery at three to seven days and more than seven days yielded VA improvement rates of 100% and 25%, respectively [15]. Wohlrab et al. reported that surgery at two days, three to seven days, and more than seven days yielded VA improvement rates of 58%, 20%, and 0%, respectively [16]. The total VA improvement rate in our study was 59.1%, which is similar to other studies. Patients with no light perception, light perception, hand motion, and finger counting demonstrated VA improvement rates of 22.2%, 25%, 20%, and 66.7%, respectively. The visual improvement rate was lower than in other research, which may be due to surgical timing. Approximately 81.8% of patients in our study underwent surgery at more than seven days after trauma and 40.9% underwent operation at >21 days, with the latest at more than two months. Many of our patients with TON also sustained closed head injury or multiorgan trauma; therefore, the evaluation and treatment of TON was often delayed until more life-threatening injuries were addressed.

The anatomy of the optic canal, ethmoid sinus, and sphenoid sinus is complex, as is the variation and pneumatization of paranasal sinus, which increases the surgical difficulty of ETOCD. Although CT reconstruction can depict 3D images, the relationship between the optic canal and the internal carotid artery cannot be depicted on the sphenoid sinus wall due to the shielding from bony structures. With advances in computer-assisted techniques, CT postprocessing images using original CT data for 3D reconstruction of bones and VR can make full use of CT for diagnosing, preoperative planning, and postoperative assessment. In our study, we used postprocessing techniques to depict the position and relationship of the optic nerve and internal carotid on the skull base. The image could adequately present the surface morphology of the medial wall of the sphenoid sinus. During surgery, we can use the images to guide recognition of the optic canal and avoid the internal carotid (Figure 1). Using the postprocessing technique, we found two cases with traumatic carotid pseudogenicity preoperatively, which is dangerous for ETOCD. Thus, the patients were referred to the neurosurgery department.

The current consensus for endoscopic nasal optic decompression includes the following: the length of surgical decompression ranges from the orbital mouth to the cranial mouth; the bony opening should expose at least one-third to two-thirds of the circumference of the nerve; if there is an obvious compression fracture or intrathecal hemorrhage of the optic nerve, the crushed bone should be completely removed, and the optic nerve sheath should be cut open to relieve the compression of the optic nerve; and if there is hemorrhage or edema in the orbital apex tissue, the orbital fascia should be fully cut open for decompression. However, slitting of the sheath may increase the risk for cerebrospinal fluid leakage, the incidence of ophthalmic artery injury, and secondary injury to the optic nerve. Consensus still lacks support from randomized controlled studies. In our study, to reduce the risk, we did not slit the nerve sheath; instead, we used punctuated optic nerve sheath splitting to relieve compression. Yu et al. reported on 96 patients with no light perception, with a total effective rate of 46.9% after ETOCD. They also used punctuated optic nerve sheath splitting in all of their patients with no light perception [17]. In our research, we measured the length and circumference of the optic canal. We found that the middle section of the optic nerve was the narrowest (*p* < 0.05), and the medial wall of the optic canal was the longest (*p* < 0.05). We assumed that decompression may not require removal of all medial wall. If we remove the length of the shortest wall on the medial wall of the optic canal, the compression may be relieved. In our research, we decompressed the optic canal according to the preoperative measurement of the length of the shortest wall. This helped reduce the risk for complications such as cerebrospinal fluid leakage. Approximately 59.1% patients exhibited improvement in VA. One patient (4.5%) developed cerebrospinal fluid leakage. No other severe complications were observed. Some researchers have reported severe complication rates for the endoscopic endonasal approach, such as cerebrospinal fluid leakage, to be as high as 7.3% [18]. Our complication rate was lower than that reported in other studies. This may have been due to the removal of the shortest length of the optic canal on the medial wall, which do not completely open the cranial mouth.

Compared with the transethmoidal approach, the transsphenoidal approach is less invasive because it preserves the uncinate process and the anterior ethmoidal sinus and part of the posterior ethmoidal sinus. The VA improvement rate is similar to the traditional approach, but the complication rate is lower. The present research had some limitations. First, the evaluation of visual function was insufficiently comprehensive and lacked the results of the postoperative visual function. Second, the number of cases in this study was limited; therefore, the results of our decompression length need more evidence to be confirmed. The surgery should be performed by skilled endoscopic physicians. Thus, in our future research, we will include more cases and perform complete visual function examinations preoperatively and postoperatively. We will also use the postprocessing technique to locate the ophthalmic artery on the optic nerve, so as to further reduce the risks involved in surgery.

## 5. Conclusions

Based on our research, we believe that ETOCD is a feasible, safe, effective, and less invasive approach for TON patients. Compared with the conventional transethmoidal approach, it has advantages including less invasiveness (preservation of the uncinate process and the anterior ethmoidal sinus) and having easier access to the optic nerve canal. Although the anatomy of the sphenoid sinus lacks definitive landmarks, the use of CT postprocessing could facilitate identification of the optic canal during surgery.

## Figures and Tables

**Table 1:** Clinical features of 22 patients with traumatic optic neuropathy.

Characteristic	Cases
*Injury part*	
Bow	7 (31.8%)
Zygoma	8 (36.3%)
Head	7 (31.8%)

*State of conscience*	
Yes	11 (50%)
No	11 (50%)

*Optic canal fracture*	
Yes	5 (22.7%)
No	17 (77.3)

*Skull base fracture*	
Yes	9 (40.9%)
No	13 (59.1%)

*Light reflex*	
Dull	11 (50%)
Lost	11 (50%)

*Eyesight*	
NLP	9 (40.9%)
LP	4 (18.2%)
HM	5 (22.7%)
CF	3 (13.6%)
<0.1	1 (4.5%)

*Time from injury to surgery*	
3–7 day	1 (4.5%)
After 7 days	21 (95.5)

*Type of injury*	
Automobile accident	6 (27.3%)
Blast	3 (13.6%)
Fall	8 (36.3%)
Assault	5 (22.7%)

**Table 2:** Comparison of the length of each wall of the optic canal.

	Group I	Group J	Mean difference (I-J)	Std. error	Sig.	95% interval confidence	
						Lower bound	Upper bound
LSD	MW	LW	2.6410	0.6433	0.0001	1.3617	3.9201
		SW	3.6795	0.6433	0.0000	2.4003	4.9587
		IW	4.6068	0.6433	0.0000	3.3276	5.8860
	LW	MW	−2.6409	0.6433	0.0001	−3.9201	−1.3617
		SW	1.0386	0.6433	0.1101	−0.2406	2.3178
		IW	1.9660	0.6433	0.0030	0.6867	3.2451
	SW	MW	−3.6795	0.6433	0.0000	−4.9587	−2.4003
		LW	−1.0386	0.6433	0.1101	−2.3178	0.2406
		IW	0.9272	0.6433	0.1532	−0.3519	2.2065
	IW	MW	−4.6068	0.6433	0.0000	−5.8860	−3.3276
		LW	−1.9659	0.6433	0.0030	−3.2451	−0.6867
		SW	−0.9273	0.6433	0.1532	−2.2064	0.3519

MD: mean difference, LSD: least significant difference, MW: medial wall, LW: lateral wall, SW: superior wall, and IW: inferior wall. The medial wall of the optic canal is the longest, which has significant difference to the superior wall, lateral wall, and inferior wall (*p* < 0.05).

**Table 3:** Comparison of the diameter of each segment of the optic canal.

	Group I	Group J	Mean difference (I-J)	Std. error	Sig.	95% interval confidence	
						Lower bound	Upper bound
LSD	Orbital mouth	Cranial mouth	−0.8386	0.1912	0.0000	−1.2208	−0.4565
		Midpiece	0.1614	0.1912	0.4019	−0.2208	0.5435
	Cranial mouth	Orbital mouth	0.8386	0.1912	0.0000	0.4565	1.2208
		Midpiece	1	0.1912	0.0000	0.6179	1.3821
	Midpiece	Orbital mouth	−0.1613	0.1912	0.4019	−0.5435	0.2208
		Cranial mouth	−1	0.1912	0.0000	−1.3821	−0.6179

The midpiece of optic canal is the narrowest, which has significant difference to the cranial mouth and orbital aperture (*p* < 0.05).

## Data Availability

The data used to support the findings of this study are included within the article.
