# Supplementary material for: Transsphenoidal Optic Canal Decompression for Traumatic Optic Neuropathy Assisted by a Computed Tomography Image Postprocessing Technique
Source: J Ophthalmol. 2020 Aug 12;2020:1870745. doi: 10.1155/2020/1870745 (PMC7441438; doi:10.1155/2020/1870745)
Supplement: Supplementary Materials — The postprocessing method is provided in the supplementary material for comprehensive parameter setting. [file 1870745.f1.docx]

**CT post-processing technique**

**CT Scans**

CT images were obtained on a 64-slice multidetector scanner (Sensation 64; GE, America). Imaging parameters for CT scanning were as follows: section thickness, 0.625 mm; tube voltage, 120 kVp; 240-300mAtube current;pitch,0.984/1; matrix, 512 × 512; scanning time,5s; contrast medium (Optiray300 mgI/mL; Coviden Japan, Tokyo, Japan); injectionrate4-5mL/s, delayed time, 18-22s.

**Post-processing**
Post-processing was performed using the software aw4.6 (GE, America),which provided axial, coronal and sagittal reconstructions of the vessels and segmentations. The post-processed images created at our institution include:

￭Fuse the Volume-rendered (VR) image of the optic nerve, the internal carotid-ophthalmic artery and overall arterial stage.

￭Rotate the VR image of overall arterial stage and expose the sphenoid sinus, adjust the sphenoid sinus VR threshold to see the mucosal surfaces of sphenoid sinus.

￭Magnify images to show the sphenoid sinus area only, and then save the VR images.

￭Do not change the angle, and adjust the overall VR display transparency to 20%, which can present the optic nerve and internal carotid artery behind the sphenoid sinus wall, and then save the image.

￭Repeat "c", "d" at different angles, and contrast the images in order to ensure the location of optic nerve and internal carotid-ophthalmic artery projected on the sphenoid sinus wall.
